# Fieber mit Exanthem nach Auslandsreise – zwei Fallberichte zu Impfversagen

**DOI:** 10.1007/s00108-026-02182-6

**Published:** 2026-09-08

**Authors:** Annika P. Schnell, Luisa Berckenhagen, Stefan Schmiedel

**Affiliations:** https://ror.org/01zgy1s35 grid.13648.38 0000 0001 2180 3484 I. Medizinische Klinik und Poliklinik, Universitätsklinikum Hamburg-Eppendorf, Hamburg, Deutschland

**Keywords:** Masern, Impfung, Immundefekt, Fieber, Reiserückkehrer, Measles, Vaccine, Immunodeficiency, Fever, Returning traveler

## Abstract

Masern treten in Deutschland vorwiegend importiert oder importassoziiert auf. Wir berichten über zwei Reiserückkehrer mit Masernimpfversagen: einen 49-Jährigen mit primärem Impfversagen nach einmaliger Impfung und schwerer Masernpneumonitis sowie einen 22-Jährigen mit sekundärem Impfversagen bei Verdacht auf Immundefekt und mildem Verlauf mit atypischem Exanthem. Masern sind auch bei Geimpften eine relevante Differenzialdiagnose und die reisemedizinische Beratung sollte den Masernimpfstatus einschließen.

## Abstract (en)

Measles cases in Germany are predominantly imported or import-associated. Two returning travelers with measles vaccine failure are reported: a 49-year-old male with primary vaccine failure after single-dose vaccination who developed severe measles pneumonitis, and a 22-year-old male with secondary vaccine failure in the context of suspected immunodeficiency who presented with a mild course and atypical exanthem. Measles remains a relevant differential diagnosis even in vaccinated individuals, and travel medicine consultations should routinely include an assessment of measles vaccination status.

## Kasuistik 1: 49-jähriger Patient mit primärem Impfversagen

### Anamnese

Die Vorstellung erfolgte als Übernahme aus einem externen Haus. Eine Woche nach Rückkehr von einer Indonesienreise entwickelte der 49-jährige Patient Fieber bis 40 °C, begleitet von einer Konjunktivitis, trockenem Husten mit Dyspnoe, Schüttelfrost, Gliederschmerzen, Diarrhö, Übelkeit und Erbrechen. Drei Tage nach Fieberbeginn trat ein makulopapulöses Exanthem auf. An Vorerkrankungen bestand lediglich eine Gicht. Der Patient war im Alter von zwei Jahren einmal gegen Masern geimpft worden.

### Befund

Bei Übernahme war der Patient initial hypoton (94/70 mm Hg), grenzwertig tachykard (100/min), febril (39,4 °C) und sauerstoffpflichtig (2–3 l/min). In der körperlichen Untersuchung imponierte ein nicht juckendes, makulopapulöses, stammbetontes Exanthem mit Beteiligung der Arme und des Gesichts (s. Abb. 1) sowie eine Konjunktivitis. In der Auskultation der Lunge hörte man basale, linksseitige, trockene Rasselgeräusche.

**Abb. 1:**
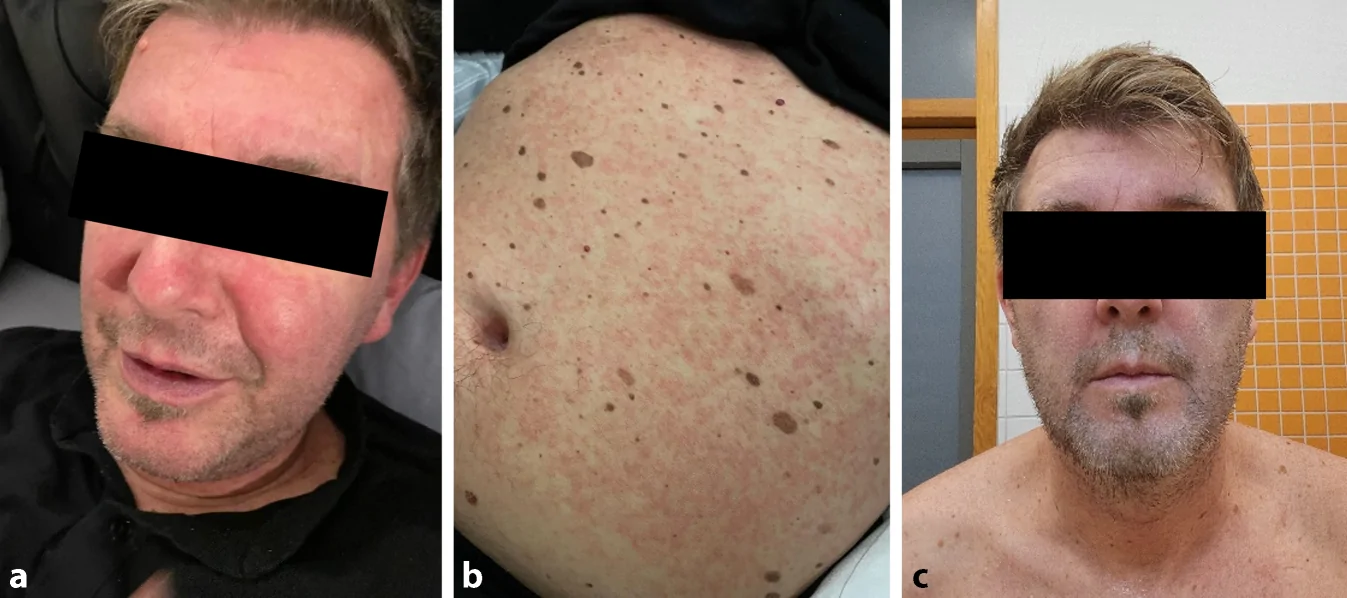
Makulopapulöses Exanthem im Gesicht (**a**) und am Stamm (**b**). Trockene, schuppige Haut nach Abklingen des Exanthems (**c**).

Laboranalytisch fielen eine leichtgradige Thrombozytopenie (136/µl), eine Erhöhung des C‑reaktiven Proteins (CRP; 118 mg/l), ein akutes Nierenversagen Grad II, eine Bilirubinämie (Gesamtbilirubin 3 mg/dl) sowie stark erhöhte Transaminasen (Alanin-Aminotranferase [ALT] 928 U/l, Aspartat-Aminotransferase [AST] 493 U/l) und Cholestaseparameter (γ-Glutamyltransferase [GGT] 734 U/l, alkalische Phosphatase [AP] 326 U/l) auf. Die Computertomographie des Thorax zeigte kleinere Konsolidierungen im linken Unterlappen und einzelne bipulmonale Noduli (s. Abb. 2a).

**Abb. 2:**
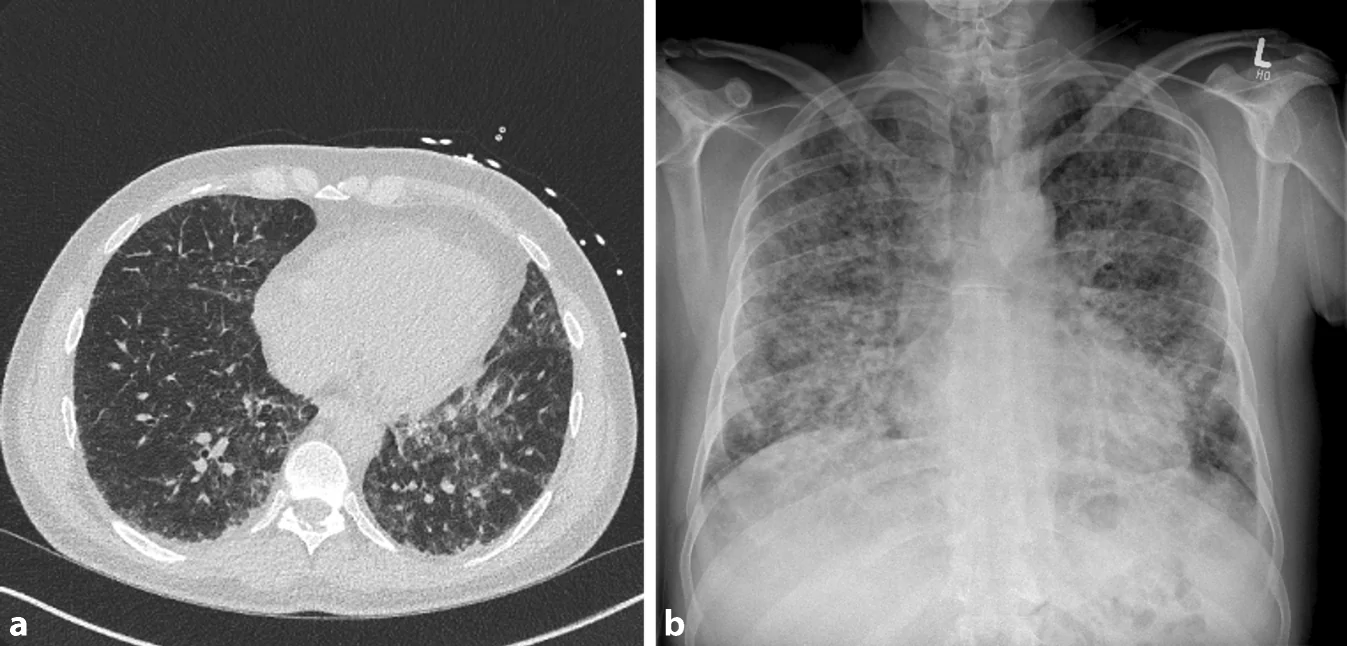
Computertomographie des Thorax (**a**) mit Konsolidierungen im linken Unterlappen und einzelnen bipulmonalen Noduli. Röntgen Thorax (**b**) vier Tage später mit ausgeprägten fleckförmigen bipulmonalen Infiltraten mit Punctum maximum im rechten Lungenunterfeld.

### Diagnose

Die initiale Masernserologie erbrachte ein positives Masern-Immunglobulin M (IgM) bei negativem Immunglobulin G (IgG). Bei nur einmaliger Impfung in der Kindheit lag ein primäres Impfversagen vor. Neben dem indirekten Masernnachweis gelang mittels Reverse-Transkriptase-Polymerase-Kettenreaktion (RT-PCR) der direkte Nachweis in Nasopharyngeal- und Konjunktivalabstrich, Urin und EDTA-Blut. Innerhalb von vier Tagen kam es zur Serokonversion mit Masern-IgG-Nachweis.

### Therapie und Verlauf

Bei dem initialen Verdacht auf eine ambulant erworbene Pneumonie oder reiseassoziierte Infektion (z.B. Leptospirose, Tsutsugamushi-Fieber) wurde eine kalkulierte antiinfektive Therapie mit Ampicillin/Sulbactam und Doxycyclin begonnen, die nach dem Masernnachweis beendet wurde. Im Verlauf stiegen Sauerstoffbedarf (bis 6 l/min) und Atemfrequenz (bis 40/min), und das Verlaufs-Röntgen (s. Abb. 2b) zeigte ausgeprägte bipulmonale Infiltrate, vereinbar mit einer Masernpneumonitis. Unter supportiver Therapie entfieberte der Patient und konnte vom Sauerstoff entwöhnt werden. Nach Regredienz des Exanthems zeigte sich die typische Hautschuppung. Das akute Nierenversagen, am ehesten prärenal, wurde mit parenteraler Volumensubstitution behandelt. Bei Entlassung hatten sich CRP, Bilirubin und Nierenfunktion normalisiert; Transaminasen und Cholestaseparameter zeigten eine Normalisierungstendenz. Der Ehefrau wurde eine zweite Masernimpfung empfohlen.

## Kasuistik 2: 22-jähriger Patient mit sekundärem Impfversagen

### Anamnese

Die Vorstellung in unserer Notaufnahme erfolgte eine Woche nach Rückkehr von einer Südafrikareise, wo der Patient Kontakt zu einem Masernpatienten gehabt hatte. Vorstellungsgrund waren Fieber mit Schüttelfrost, Nachtschweiß und Arthralgien sowie im Verlauf Diarrhö, ein stammbetontes Exanthem, trockener Husten und Brennen der Augen. Der Patient war in der Kindheit zweimal gegen Masern geimpft worden. Einige Monate zuvor war als Zufallsbefund in einer Serumeiweißelektrophorese eine relative Erniedrigung der Gammaglobulinfraktion aufgefallen und ein Immundefekt vermutet worden. Eine erhöhte Infektanfälligkeit sowie andere Vorerkrankungen wurden verneint.

### Befund

In der körperlichen Untersuchung imponierten ein stammbetontes, makulopapulöses Exanthem (s. Abb. 3) mit geringfügiger Beteiligung des Gesichts und eine leichte Konjunktivitis. Laboranalytisch zeigten sich eine mittelgradige Leuko- und Thrombozytopenie (Leukozyten 2,9/µl, Thrombozyten 81/µl), eine milde CRP-Erhöhung (20 mg/l) sowie eine Erhöhung der Transaminasen (ALT 142 U/l, AST 101 U/l) und der Cholestaseparameter (GGT 250 U/l, AP 171 U/l). Sämtliche Immunglobulinsubklassen waren erheblich erniedrigt.

**Abb. 3:**
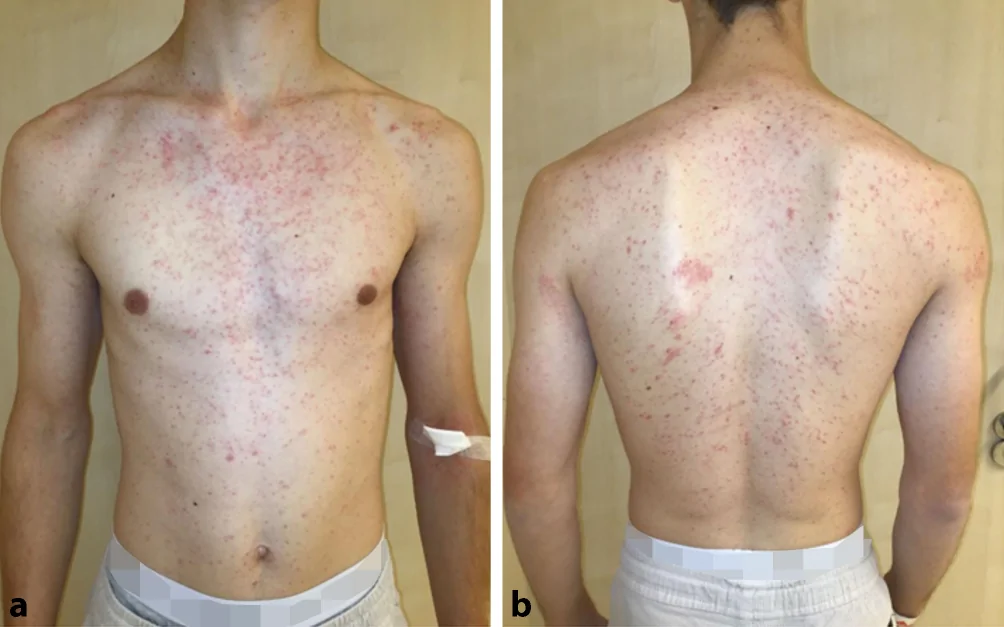
Atypisches makulopapulöses Masernexanthem am Stamm (**a**, **b**) bei sekundärem Impfversagen.

### Diagnose

Mittels RT-PCR konnte Masernvirus im Nasopharyngeal- und Konjunktivalabstrich sowie Urin nachgewiesen werden. Serologisch zeigte sich Masern-IgG ohne IgM-Antwort, entsprechend einem sekundären Impfversagen bei zweimaliger Impfung in der Kindheit.

### Therapie und Verlauf

Unter supportiver Therapie kam es zur spontanen Besserung der klinischen Symptomatik und der Laborparameter. Die Leukozytopenie wurde infektassoziiert gewertet. Aufgrund des ausgeprägten Immunglobulinmangels mit Pan-Hypogammaglobulinämie (IgG 2,47 g/l, Norm 6,5–16 g/l; IgA 0,20 g/l, Norm 0,4–3,5 g/l; IgM 0,09 g/l, Norm 0,5–3 g/l) erfolgte die Überweisung zur ambulanten Immundefektabklärung.

## Diskussion

Die hier berichteten Masernfälle (s. Tab. 1) charakterisieren das primäre und sekundäre Impfversagen. Beim ersten Patienten, der lediglich eine Impfung erhalten hatte, kam es mutmaßlich nicht zur Ausbildung einer Immunantwort. Alternativ könnte das initial negative Masern-IgG eine bereits abgeklungene Immunität widerspiegeln. Eine einmalige Impfung hat eine Wirksamkeit von ca. 95 %. Die zweite empfohlene Impfung dient dem Schließen von Immunitätslücken [1] und hätte möglicherweise den schweren Verlauf mit Masernpneumonitis verhindert. Die STIKO empfiehlt daher allen nach 1970 Geborenen mit keiner oder nur einmaliger Impfung bzw. unklarem Impfschutz eine zweite Dosis des Masern-Mumps-Röteln-Impfstoffs [2].

**Tab. 1:** Patientencharakteristika.

	Fall 1 (♂, 49)	Fall 2 (♂, 22)
Impfstatus Masern	1× in der Kindheit	2× in der Kindheit
Reiseland	Indonesien	Südafrika
Vorerkrankungen	Gicht	V. a. Immundefekt
Direkte Diagnostik	RT-PCR pos. in Nasopharyngealabstrich, Konjunktivalabstrich, Urin, Blut	RT-PCR pos. in Nasopharyngealabstrich, Konjunktivalabstrich, Urin
Indirekte Diagnostik	IgM pos., IgG neg. (Serokonversion nach vier Tagen)	IgM neg., IgG pos.
Exanthem	Typisch (makulopapulös, großflächig, konfluierend)	Atypisch (schwächer, nicht konfluierend)
Komplikation	Masernpneumonitis, akutes Nierenversagen Grad II	–
Impfversagen	Primär	Sekundär

*♂* männlich, *RT-PCR* Reverse-Transkriptase-Polymerase-Kettenreaktion, *pos.* positiv, *IgM* Immunglobulin M, *IgG* Immunglobulin G, *neg.* negativ

Beim zweiten Patienten verhinderte eine bestehende Restimmunität eine schwere Erkrankung und führte zu einem abgeschwächten, „mitigierten“ Verlauf mit atypischem Exanthem: schwächer ausgeprägt, stammbetont, ohne Konfluenz. Das sekundäre Impfversagen war am ehesten durch den zugrunde liegenden Immundefekt bedingt.

Der klinische Verdacht auf Masern sollte labordiagnostisch bestätigt werden. Die alleinige Masernserologie wird nicht mehr empfohlen. Der zweite Fall wäre damit verpasst worden. Besser geeignet ist die RT-PCR aus Rachenabstrich (positiv bis Tag 7) oder Urin (Tag 3–10; [3]). Auch Nachweise in anderen Körperflüssigkeiten wie hier Blut können positiv sein.

Der zweite Fall illustriert zudem die Bedeutung der Herdenimmunität. Sie schützt Personen, die nicht geimpft werden können, wie Säuglinge, Schwangere und Immundefiziente, oder keine ausreichende Immunität ausbilden können. Eine Bevölkerungsimmunität von ≥ 95 % ist zur Masernelimination erforderlich [3]. Deutschland verfehlt dieses Ziel bislang [4]. Hinzu kommt, dass eine Maserninfektion selbst einen passageren Immundefekt für Wochen bis Monate verursachen kann, unter anderem vermutlich durch Verdrängung des Gedächtniszellrepertoires [5].

Nach dem Ende der COVID-19-Pandemiemaßnahmen kam es in Deutschland wieder zu einem Anstieg der Masernfälle, sodass 2024 wieder das präpandemische Niveau erreicht wurde. Mindestens 82 % der Fälle waren dabei aus dem Ausland importiert oder importassoziiert [4]. Die Masernimpfung spielt somit auch im reisemedizinischen Kontext eine wichtige Rolle. 51 Länder, darunter Indonesien und Südafrika, erfüllen aktuell die WHO-Kriterien für einen großen Masernausbruch [6, 7]. Im Rahmen der reisemedizinischen Beratung sollte daher auch die Vollständigkeit der Standardimpfungen, inkl. der Masernimpfung, geprüft werden [8].

Wir berichten hier von einem Fall mit einer schweren Masernpneumonitis bei primärem Impfversagen und einem atypischen Exanthem bei sekundärem Impfversagen. Die beiden Fälle unterstreichen die Notwendigkeit, bei Fieber und Exanthem auch an Masern zu denken, selbst bei geimpften Erwachsenen, bei denen Verlauf und Exanthem atypisch sein können.

## Fazit für die Praxis

- Auch bei ein- oder zweimaliger Masernimpfung stellt eine Maserninfektion bei Fieber unklarer Genese mit Exanthem eine relevante Differenzialdiagnose dar.
- Bei vorausgegangener Impfung kann sich das Masernexanthem atypisch darstellen.
- Standardimpfungen wie Masern sollten im Rahmen einer reisemedizinischen Beratung berücksichtigt und gegebenenfalls vervollständigt werden.

## Data Availability

Alle den Ergebnissen zugrunde liegende Daten sind im Artikel enthalten. Weitere Daten sind auf begründete Anfrage bei der Erstautorin erhältlich.
